# Tumor organoid biobank-new platform for medical research

**DOI:** 10.1038/s41598-023-29065-2

**Published:** 2023-02-01

**Authors:** Xuexue Xie, Xinyu Li, Wei Song

**Affiliations:** 1 First College of Clinical Medicine, Shandong University of Traditional Chinese Medicine, Jinan, Shandong People’s Republic of China; 2 Department of Minimally Invasive Comprehensive Treatment of Cancer, Shandong Provincial Hospital Affiliated to Shandong First Medical University, Jinan, Shandong People’s Republic of China; 3 Department of Minimally Invasive Comprehensive Treatment of Cancer, Shandong Provincial Hospital Affiliated to Shandong First Medical University, No.324, Jingwuweiqi Road, Jinan, 250021 People’s Republic of China

**Keywords:** Cancer, Medical research, Oncology

## Abstract

Organoids are a new type of 3D model for tumor research, which makes up for the shortcomings of cell lines and xenograft models, and promotes the development of personalized precision medicine. Long-term culture, expansion and storage of organoids provide the necessary conditions for the establishment of biobanks. Biobanks standardize the collection and preservation of normal or pathological specimens, as well as related clinical information. The tumor organoid biobank has a good quality control system, which is conducive to the clinical transformation and large-scale application of tumor organoids, such as disease modeling, new drug development and high-throughput drug screening. This article summarized the common tumor types of patient-derived organoid (PDO) biobanks and the necessary information for biobank construction, such as the number of organoids, morphology, success rate of culture and resuscitation, pathological types. In our results, we found that patient-derived tumor organoid (PDTO) biobanks were being established more and more, with the Netherlands, the United States, and China establishing the most. Biobanks of colorectal, pancreas, breast, glioma, and bladder cancers were established more, which reflected the relative maturity of culture techniques for these tumors. In addition, we provided insights on the precautions and future development direction of PDTO biobank building.

## Introduction

Tumor is a heterogeneous disease^1–5^, which seriously threatens human life and health. In recent years, substantial progress has been made in immunological and targeted therapy for malignant tumors. The main obstacle to the development of new drugs is the clinical translation of scientific results, and the key to overcoming this obstacle is the selection of high-quality preclinical research models^6,7^. Existing research models, such as immortalized cell lines, patient-derived tumor xenografting (PDX), and animal models, have their own advantages and disadvantages (Table 1). Immortalized cell lines can be gene edited and allow high-throughput drug screening, with the advantages of easy access and strong experimental reproducibility. But most tumor cell lines have lost the heterogeneity of primary tumors after long-term selective culture in a single environment in vitro. Another preclinical model is PDX, which can mimic tumor heterogeneity and microenvironment, but is very costly, time-consuming, and has a low success rate. Although animal model established by injecting tumor cell lines or tumor cell spheres into immunodeficient mice can create a relative in vivo environment, it may still affect the accuracy of the experiment due to species differences or lack of immune microenvironment. Therefore, researchers are committed to developing a preclinical model that can truly reflect the characteristics of patients, and organoids have been widely recognized by the medical community as a new type of 3D model. Tumor organoids are formed by mechanically and enzymatically extracting tumor cells from fresh tumor tissue and cultured them in specific matrices, which can reflect the heterogeneity of parental tumor tissue^8–10^. PDO are high-quality model for preclinical research. Many of the major discoveries in basic and translational medicine benefited from exploratory research on large biobanks^11–15^. At present, many academic and commercial groups have established their own PDTO biobanks, but how to standardize quality control of biobank still needs a unified standard. The establishment of standards will help the promotion of organoid models. This article summarized the current development status of PDTO biobanks and provided insights on the precautions and future development direction.

**Table 1:** Comparison of preclinical patient-derived tumor research models.

Models	Immortalized cell line	Tumor spheres	PDX	PDTO
Cultivating way	2D	3D	3D	3D
Maintenance and passage	Easy	Moderate difficulty	Difficult	Moderate easy
Growth speed	Rapid	Rapid	Slow	A little slow
Heterogeneity of tumor	Lack	Lack	Preserve	Preserve
Tumor microenvironment	Lack	Lack	Partly retention	Partly retention
Established success rate	Low	Low	Slightly lower	Moderate
Cost	Low	Slightly higher	High	Slightly higher
Gene editing	Editable	Editable	Not editable	Editable
High-throughput drug screening	Allow	Allow	unallowed	Allow
Large scale application	Allow	Not recommended	unallowed	Allow
Normal controls	None	None	None	Have
Genetic tumor model	None	None	Have	Have

## Overview of PDTO biobanks

As a novel 3D culture model, organoids^16–18^ are in vitro miniature organs composed of epithelial cells. Sato et al.^19^ first proposed that LGR5 + mouse intestinal stem cells can proliferate indefinitely through organoids. There is increasing evidence that PDTOs retain the histological and genomic characteristics^20–25^ of parental tumor for use in personalized medicine^26^ and new drug development^27–30^, with great potential compared to other models^31^. In basic research, exon and transcriptome sequencing analysis can be used to find key mutations and transcriptome changes, which is helpful to further explore the mechanism of tumor genesis, development and treatment resistance or sensitization^32–34^. The PDTO biobanks centrally manage and utilize organoid information. Since Sato et al. developed the first organoid model^19^ from mouse small intestine in 2009, substantial progress has been made in the field^35–37^. At present, many commercial and academic groups have established their own PDTO biobanks. Here, we summarized the current PDTO biobanks and related information (Additional file 1).

### Comprehensive PDTO biobanks

Organoids, as "patients in the laboratory", can better reflect tumor heterogeneity and drug response. The organoid biobank, established by the Hubrecht Institute, Utrecht University Medical Center and the Royal Netherlands Academy of Arts and Sciences is one of the most comprehensive organoid biobanks. It collected more than 1,000 organoids from a variety of organs and diseases, including breast, colon, head and neck tumors, intestinal, liver, lung, ovarian, and pancreatic tumors, as well as a large number of intestinal organoids in cystic fibrosis patients^38,39^. In addition, organoid biobank established by commercial groups such as Sigma-Aldrich, the American Typical Culture Collection, Cellesce and DefiniGEM stored large amounts of PDOs from induced pluripotent stem cells (iPSCs) or primary tissues, covering healthy individuals and patients. Details of organoid culture were also available. Some organoid lines were derived from iPSCs, expanding the sources of organoid and their potential applications.

### Brain, head and neck PDTO biobanks

Brain tumors were the 10th leading cause of death in 2020^40^. Glioblastoma (GBM) is the most common primary malignant brain tumor in adults, which has the lowest 1-year survival (40.9%) and 5-year survival (6.6%) compared to other primary brain tumors^41–43^. For more than a decade, the standard of care has been surgical resection combined with chemotherapy and radiotherapy. Heterogeneity between tumors^44^ and within tumors^45–47^ can lead to poor outcomes in many clinical trials^48^. Therefore, there is an urgent need for reliable preclinical research models that can adequately reflect tumor heterogeneity. Jacob et al.^12^ reported a method to culture PDTOs directly from fresh brain tumor tissue without single-cell dissociation. Histological, molecular, and genomic analyses had found glioma organoids preserved key features of parental tumors, which could be used to predict therapeutic efficacy. In addition, Abdullah et al.^49^ established a PDTOs biobank of low-grade gliomas that preserved the molecular and histological features of primary tumors. Importantly, the organoids they cultured retained a diverse cellular environment, enabling future studies of the glioma microenvironment.

Head and neck tumors include neck tumors, otolaryngology tumors, oral and maxillofacial tumors. Although the 5-year overall survival rate of nasopharyngeal carcinoma is higher than 80%^50^, 10% ~ 15% of patients have tumor recurrence, and the 5-year overall survival rate is only 13.2% ~ 38%^51,52^. The high recurrence rate is responsible for the poor prognosis for most nasopharyngeal cancers^53^. Wang et al.^54^ established a PDTO biobank of 39 primary and recurrent nasopharyngeal carcinomas and found that all nasopharyngeal carcinomas carried Epstein-Barr virus and kept the virus expanding. Furthermore, their stem cell markers were expressed more in recurrent organoids than in primary. It can be seen that PDTOs can be stably passaged and contribute to the study of tumorigenesis and development.

### Digestive PDTO biobanks

Some research groups have established their own PDTOs biobanks, with organoid lines ranging from dozens to hundreds. Hans Clevers' team first established the colorectal cancer organoid biobank in 2015, who successfully cultivated 22 colorectal cancer organoids with an overall success rate of 90%, and the survival rate after resuscitation can reach more than 80%^20^. Vlachogiannis et al.^21^ collected 110 fresh tissues from 71 patients to establish a PDTO biobank derived from patients with metastatic, post-treatment colorectal and gastroesophageal cancers. Their PDTOs could also be established with a low tumor/stromal ratio. Chinese scholars^55^ also established a ‘paired organoid’ biobank from 20 microsatellite stable early-onset colorectal cancer patients. They found that R-Spondin fusion organoids were similar to normal colon organoids, with a tendency to mature when Wnt withdraws, while Adenomatous Polyposis Coli mutant organoids were locked in the progenitor cell stage. Yao et al.^56^ established a biobank from locally advanced rectal cancer patients receiving neoadjuvant chemoradiotherapy (NACT). They used organoids to predict the response to chemoradiotherapy with an accuracy of 84.43%, a sensitivity of 78.01%, and a specificity of 91.97%. Geevimaan et al.^57^ validated the efficacy of oxaliplatin as first-line adjuvant chemotherapy for advanced colon cancer by organoids. They found that oxaliplatin-sensitive and drug-resistant patients were two separate populations, and genomics had identified 18 genetic signatures that predicted drug response. Mo et al.^58^ established a biobank of 50 primary colon cancer and paired liver metastases, and multi-omics analysis confirmed that organoids can reflect the tumor. Laoukili et al.^59^ collected intraoperative ascites from patients with peritoneal metastases of colon cancer, generating a biobank composed of 35 primary tumor regions and 59 paired peritoneal metastases from 12 patients. They found that Consensus Molecular Subtype 4 improved the sensitivity to oxaliplatin by inhibiting reducing ability. Other research teams^60–62^ also found that PDTOs reflected tumor heterogeneity. It is demonstrated the advantages of PDTO including high-throughput drug screening and precision medicine. Usui et al.^63^ found that Hedgehog signaling inhibitors (AY9944, GANT61) in combination with 5-FU, irinotecan or oxaliplatin reduced the cell viability of PDTO. Yan et al.^64^ established a comprehensive primary gastric cancer organoid biobank that included tissue samples from 34 patients with normal, dysplastic, tumor, and lymph node metastases. The biobank provided detailed whole exome and transcriptome analysis, providing a source of samples for studying gastric cancer development and metastasis.

Hepatocellular carcinoma (HCC) is the most common primary liver cancer, and is the second most common cause of cancer-related death worldwide^65^. The treatment currently available for HCC is not satisfactory. Nuciforo et al.^66^ successfully cultured PDTOs from puncture biopsies of HCC patients, which reproduced the histological features and maintained the genomic features over a long-term culture course of up to 32 weeks. Long-term stable passage of organoids helps to reproduce scientific experiments.

Pancreatic cancer is a highly aggressive malignancy that ranks seventh in the world for death^67–71^. New treatments are urgently needed to improve survival. Intraductal papillary mucinous neoplasm (IPMN) is a precursor to cystic pancreatic cancer^72,73^. Beato et al.^74^ established a paired organoid biobank of IPMN, in which organoids could also be generated from specially treated frozen tissue. The tissue was placed in a cryovial containing 1 ml of freezing solution and placed on ice for 30 min, then stored at -80 °C overnight prior to liquid nitrogen storage. For organoid culture, the tissue was thawed 50% at 37 °C and then in a dish for subsequent manipulation. Since there are no commonly used and well-defined methods for cryopreservation and resuscitation of tissue, culturing organoids from frozen tissue is difficult and rare. Another research team established an IPMN organoid biobank from 7 normal pancreatic ducts and 10 unpaired tumor samples and then performed molecular characterization validation^75^. Hirt et al.^76^ established a human pancreatic cancer organoid biobank covering representative subtypes and developed an automated screening process for organoid culture, drug delivery. In addition, other research teams^77,78^ also have established pancreatic cancer organoid biobanks. Vaes et al.^79^ established a pancreatic cancer organoid biobank from patients with cachexia and non-cachexia. They found PDTOs expressed a variety of cachexia-related genes, including interleukin (IL)-6, tumor necrosis factor-α, IL-8, IL-1α, IL-1β, monocyte chemoattractant protein-1, growth differentiation factor 15 and leukemia inhibitory factor. This provided a valuable tool for studying the driving mechanisms of cancer cachexia.

Gastroenteropancreatic neuroendocrine neoplasms are deadly but understudied disease. Kawasaki et al.^80^ established a biobank containing 25 organoid lines of neuroendocrine tumors. They established the first functional gastrin tumor model. Whole genome sequencing (WGS) showed frequent genetic alterations in the tumor suppressor gene tumor protein p53 (TP53) and retinoblastoma susceptibility gene. Knocking out the above two genes or overexpressing key transcription factors conferred a normal organoid phenotype. It can be seen that organoids can combine genetics and biological phenotypes to deepen the genetic understanding of disease.

### Respiratory malignancies PDTO biobanks

Lung cancer is the most common malignancy and the first cause of cancer-related death worldwide^81^. The main causes of high mortality are drug resistance and ineffective clinical drug design^82,83^. Cisplatin has been found to show higher half maximal inhibitory concentration in PDTOs derived from non-small cell lung cancer (NSCLC) compared to cell lines^84^. Therefore, organoid-based drug screening may provide a more precise therapeutic direction for clinical drug treatment. Kim et al.^85^ established 84 PDO lines from patients with advanced lung adenocarcinoma. WGS and RNA sequencing analysis found that PDTOs largely retained patients' somatic changes, especially driver gene changes. In addition, they identified new molecular targets using organoids, which reflected the value of PDTOs in translational medicine. Li et al.^86^ established an organoid biobank from 10 patients with NSCLC, 9 of whom had epidermal growth factor receptor (EGFR) mutations. Compared to natural medicines with that of cell lines (H1299, H460, and H1650), they found that PDTOs were sensitive to berberine while cell lines showed resistance. This reflects PDTOs are expected to become the next generation of preclinical tumor research models which are worth replicating.

### Urological PDTO biobanks

Kidney tumors are one of the most common solid tumors in children and lack preclinical research models that capture tumor heterogeneity. The PDTO biobank established by Calandrini et al.^87^ contained more than 50 tumors with different renal cancer subtypes and matching normal renal organs, including Wilms tumor, malignant rhabdomyoma, renal cell carcinoma, and congenital mesodermal nephroma. This is also the first pediatric PDTO biobank. It can be seen that the establishment of kidney cancer organoid biobanks is very difficult.

Most bladder cancers are urothelial cancers, most of which are non-muscle-invasive bladder cancers^88–90^. It is characterized by high morbidity, recurrence rate and treatment cost. Lee et al.^91^ established a biobank of bladder cancer organoids derived from primary tumors or multiple recurrences, fully reflected tumor heterogeneity. Weber et al.^92^ built a biobank of bladder cancer organoids, which was established from patients with low-grade non-muscle-invasive or high-grade muscle-invasive carcinoma. An organoid biobank from 53 patients with bladder cancer was also established, and found that common bladder cancer mutations, such as TP53 and Fibroblast Growth Factor Receptor 3^93^. Currently, very few organoid biobanks of bladder cancer are established. Researchers have successfully generated normal and neoplastic organoids from dog urine samples^94,95^.

Prostate cancer (PCa) is the second most common cancer in men worldwide. Common treatment modalities are drugs plus androgen deprivation therapy. However, castration resistance is prone to occur. Once it occurs, the survival time from the beginning of progression is generally 2–3 years^96^. Beshiri et al.^97^ established a biobank derived from PCa puncture samples and contained 4 castration resistance organoid lines from 3 patients. They found that a high proportion of castration-resistant tumor cells required p38 activity to optimally establish and reproduce in vitro. At present, the establishment of urinary tumor organoid biobanks is very few, which shows that the establishment of urinary tumor organoid lines is very difficult.

### PDTO biobanks for genital systems and other tumors

Cervical cancer is a common gynecologic malignancy that lacks human-derived culture systems. Lõhmussaar et al.^98^ established an organoid culture protocol for long-term culture of normal and Pap brush tumor tissue. The PDTO they cultured preserved the pathogenic human papillomavirus genome. Normal organoids can be driven malignant transformation^99,100^ by introducing oncogenic mutations using CRISPR-Case9 editing. It is very promising for organoids to explore carcinogenic mechanisms.

Ovarian cancer, particularly high-grade serous ovarian cancer, is the leading cause of gynaecological-related death^101^. Not only is it limited in treatment options, but it is also prone to relapse. Nelson et al.^102^ cultured organoids from puncture or ascites samples from patients who were naïve and retreated for ovarian cancer. They built a biobank containing 76 various PDTO lines. The culture success rate was 26.2%. They can be cultured successfully from frozen post-digested cells, which provides us with new ideas for improving the success rate of organoid culture.

Breast cancer is the most common and second deadliest malignancy in women^103^. Currently, the standard treatment of breast cancer is based only on clinical and pathological features, such as estrogen receptor (ER), progesterone receptor (PR), and human epidermal growth factor receptor 2 (HER2)^104^, which is not sufficient to achieve precise individualized treatment. Dekkers et al.^105^ provided an optimized protocol for culturing organoids from human breast cancer. The PDTO biobank they established covered the major pathological subtypes of breast cancer (triple-negative, ER + /PR + and HER2 +). Sachs et al.^106^ generated more than 100 primary and metastatic breast cancer organoids using their own culture protocol. Breast cancer organoids matched the histopathology of the parent tumor. DNA copy number changes and sequence changes were largely preserved even after long-term passage. Other research teams established breast cancer organoid biobanks^107,108^ after NACT and found that PDTOs can predict patients' response to NACT. As a special subtype of breast cancer, triple-negative breast cancer (TNBC) has fewer treatment options than other subtypes. Kim et al.^109^ explored the pathway activity, microenvironment and clinical relevance of TNBC in their own biobank, and systematically proposed a predictive model and prognostic markers. Another tumor organoid biobank of TNBC provided comprehensive genome, transcriptome, and cellular characterization^110^. TNBC organoids lost the characteristics of normal breast PDO and were mostly rich in luminal progenitor cells. The differences between normal and tumor organoids facilitate the study of tumorigenesis mechanisms.

## The key to building PDTO biobanks

### Quality control of PDTO biobank management system

**Figure 1:**
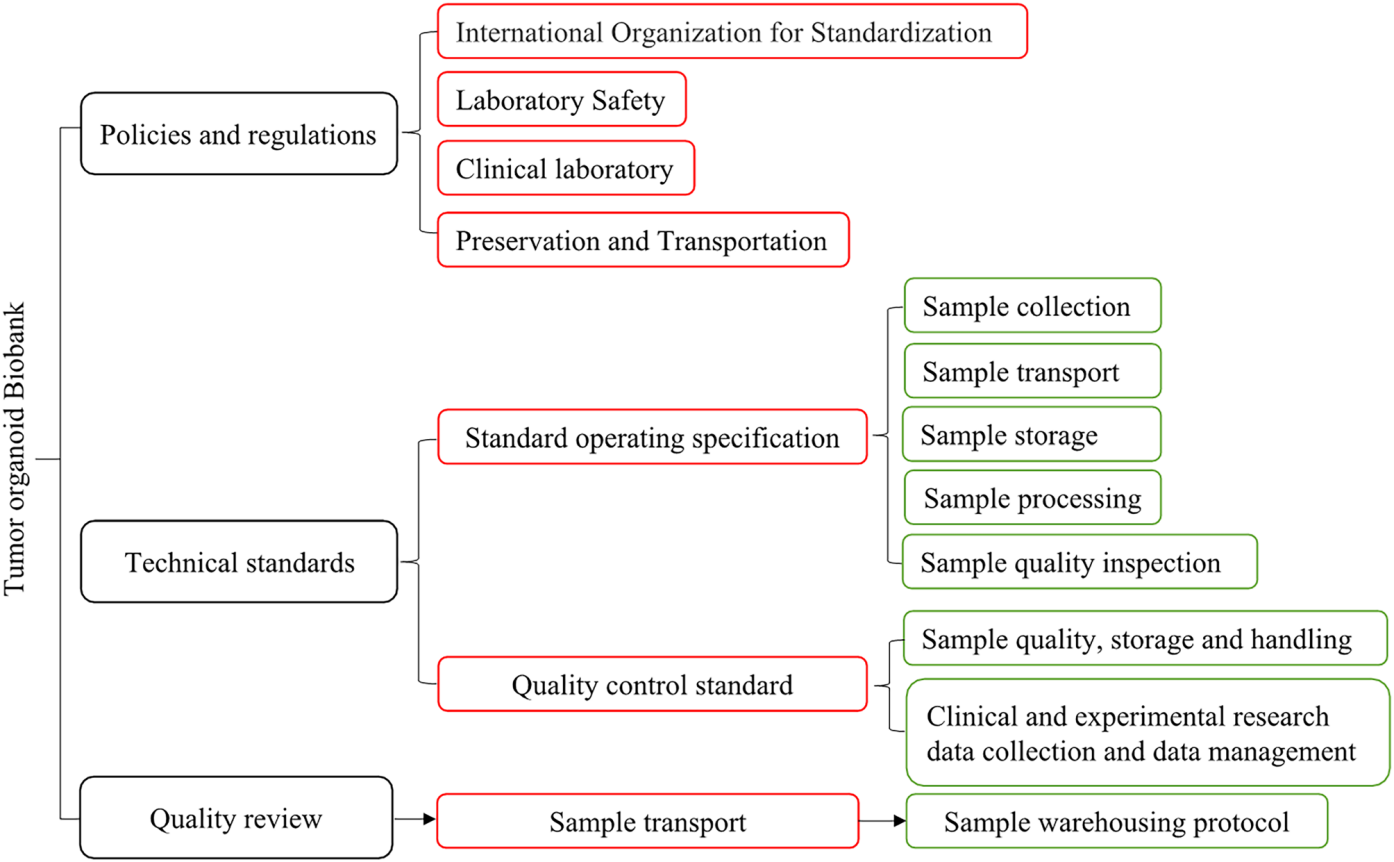
Management process and quality control system of organoid biobanks.

PDTOs can be expanded for a long time and allow cryostorage, which create conditions for the establishment of biobanks. Organoid biobanks can be shared by global researchers, which is important especially for researchers who do not have easy access to human tissue samples. Authentic, complete and traceable high-quality sample data play a critical role in the accuracy and reliability of clinical research. The basic criteria for establishing a biobank are safety, accuracy and convenience. Information related to biobank activities, processes and procedures should be documented in an easy-to-understand format. In addition, dedicated personnel are required to manage and regularly test biobank for contamination by sampling. It can be seen that the establishment of biobanks requires a complete set of management processes (Fig. 1). The contents of the management system include policies and regulations, experimental techniques and sample management. Thus, establishing PDTO biobanks is a time-consuming and labor-intensive project. Once established PDTO biobanks, they can provide reliable and accurate preclinical models for clinical research, which is conducive to large-scale exploratory research.

### Quality control of organoid culture processes

**Figure 2:**
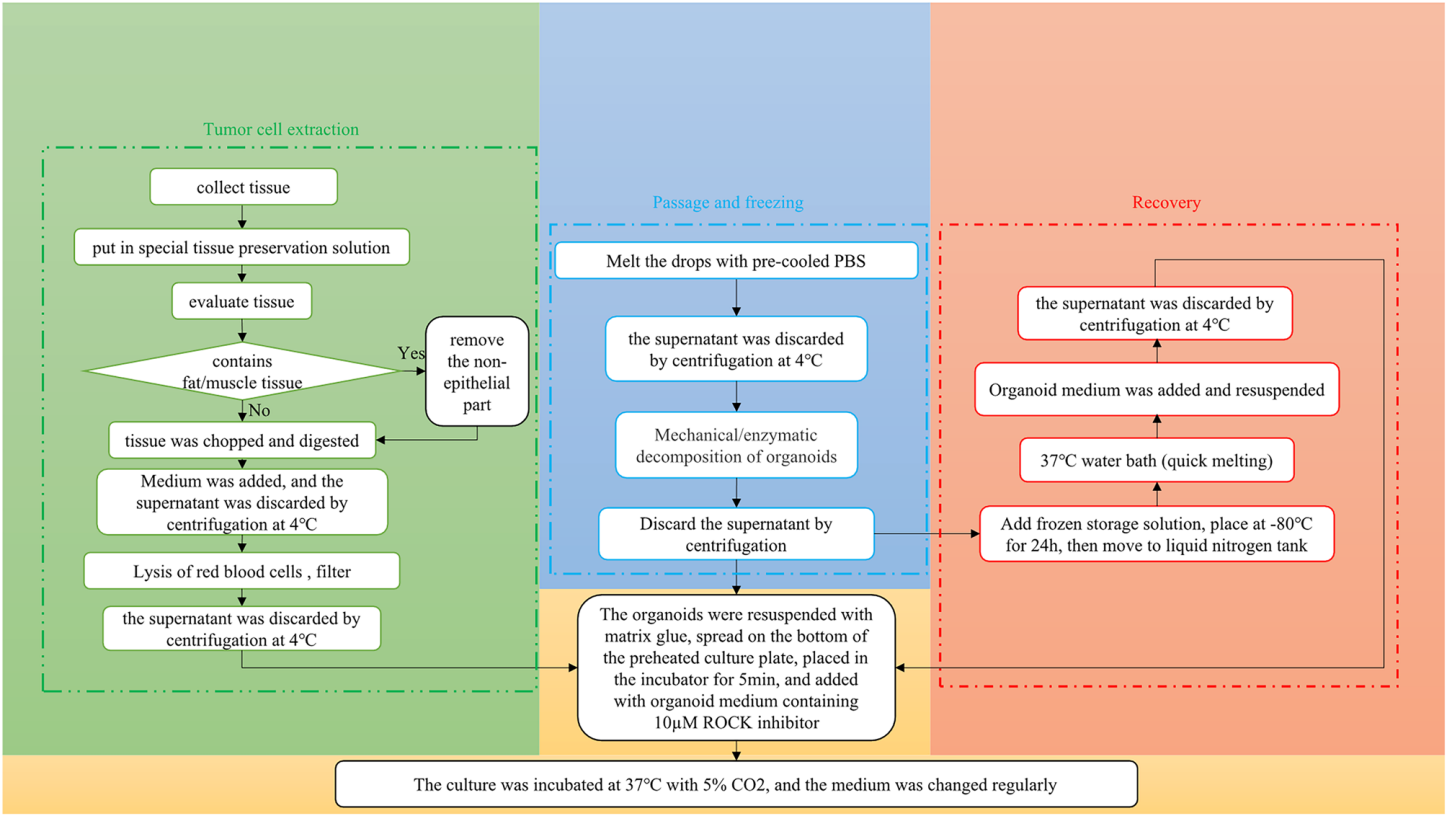
Culture process of tumor organoids. From left to right, tumor cell extraction, as well as organoid passage, cryopreservation and resuscitation.

The success rate of tumor organoid culture and resuscitation is very important for the establishment of biobanks. Therefore, quality control of the PDTO culture process (Fig. 2) has been a top priority. The acquisition of fresh tissue is the first step. According to our experience, fresh tissue should be placed in preservation solution and transported to the laboratory on ice as soon as possible. In principle, fresh tissues can be stored in the preservation solution for three days, but the ischemic time should be minimized. The longer the time, the worse the cell activity, as well as the lower the success rate of culture. After the tissues are transferred to the laboratory, they will be washed in PBS or cleaning medium with antibiotics. The number of washes is increased or decreased according to the cleanliness of the tissue. Non-epithelial parts such as fat and muscle should be first removed from tissues. Tumor cells are generally obtained under the dual action of mechanical and enzymatic digestion. Impurities are removed by manipulations such as red blood cell lysis and strain. Cells are subsequently resuspended using a matrix (usually Matrigel or BME). After planting plates, organoids are cultured using specific amplified medium. It is necessary to change the solution every 2 ~ 4 days. Organoids can form in 3 ~ 5 days after being seeded in plate, and can be passaged after 7 ~ 14 days. The passage ratio is determined according to the density of organoids. PDTOs have morphological diversity, which can be solid, cystic or mixed. Triple Express or Cell Recovery Solution is usually used for solid organoids. Combined with mechanical force, organoids can be dispersed into small fragments or single cells. Organoids are stored in cryopreservation solution at − 80 °C overnight and then transferred to liquid nitrogen for long-term storage. In addition, the success rate of organoid resuscitation is also very important. The principle of slow freezing and thawing should be followed, just like cell lines. After resuscitation, PDTOs need to grow in 1 ~ 2 weeks to restore their expansion capacity.

Some problems (Additional file 1) exist in the culture process of organoids, such as the way to overcome the contamination of normal organoids in PDTOs. In addition to selecting PDTOs by adding and subtracting cytokines in culture medium, it can also be selected under the microscope. Careful observation is critical in the process of organoid culture. Attention should be paid to avoid contamination within three days after organoids being planted in plates, and timely treatment should be given to avoid cross-contamination. In general, organoid culture has many detailed requirements, such as mechanical force, digestion time, seeding density. Errors in any procedure may lead to experimental failure.

## Application of tumor organoid biobanks

**Figure 3:**
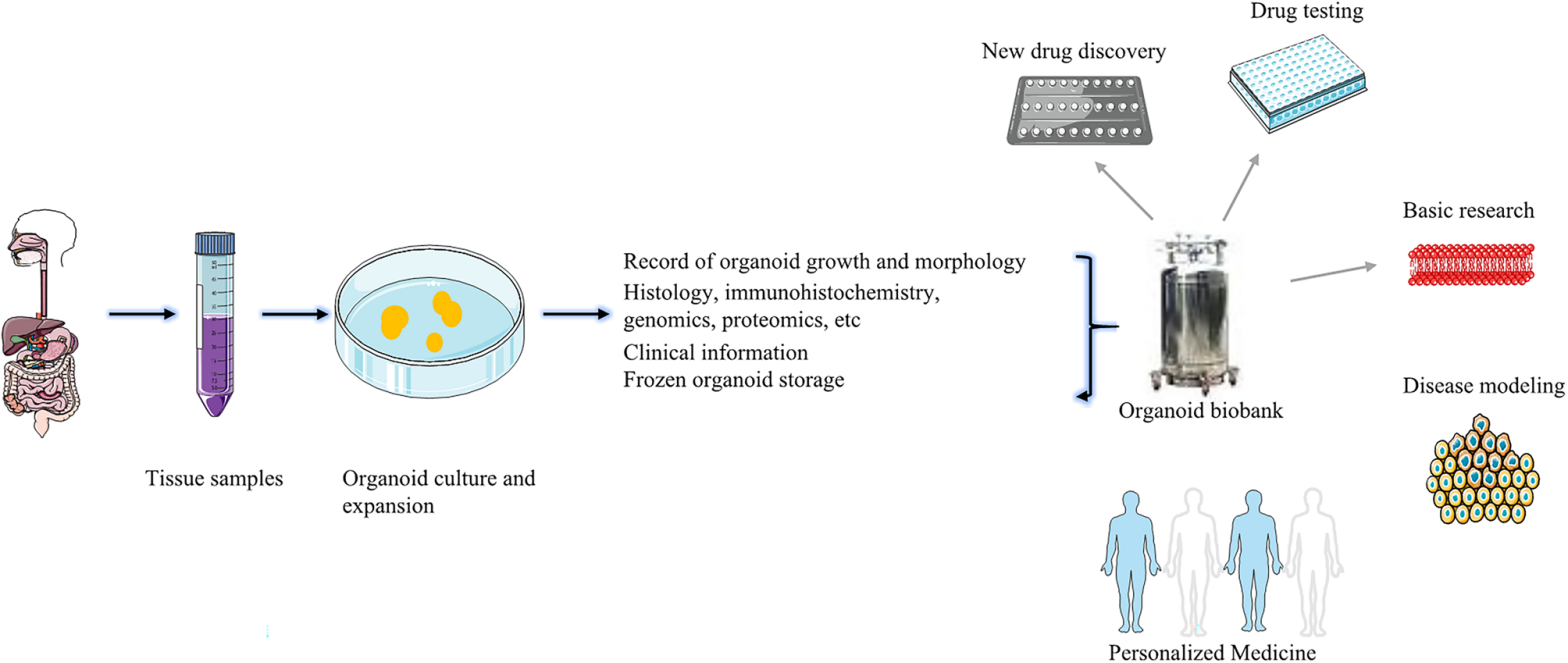
Establishment and application of tumor organoid biobank.

Most new anti-cancer drugs cannot be successfully used in clinic. The key factor is lack of effective preclinical models in the early research and development (R&D) process. As a new type of preclinical research 3D model, previous studies have demonstrated that PDTOs can reflect the phenotypic characteristics of parental tumor tissue, suggesting that organoid have great potentials in drug screening^111–115^. Some researchers have also established a biobank of PDTOs for colon cancer to find biomarkers that can predict the efficacy of EGFR inhibitors^116,117^. This suggests that PDTO biobanks are suitable for new drug development and high-throughput drug screening. Most organoid culture and intervention experiments can be completed within 4 weeks, indicting that PDTOs can make meaningful guidance for clinical medication in a short period of time. For patients who are not sensitive to chemoradiotherapy, PDTOs can not only help find new therapeutic targets, but also conduct basic research. Yu et al.^118^ found that inhibition of human specific endogenous retrovirus H prevented the growth of colon cancer cells and PDTOs. It has been demonstrated that fibrillin-1/vascular endothelial growth factor-2/ signal transducer and activator of transcription 2 signaling pathway can induce chemotherapy resistance in ovarian PDTOs by participating in glycolysis and angiogenesis^33^. It can be seen that the establishment of PDTO biobanks is conducive to explore the mechanism of tumorigenesis and development. It helps to discover new anti-cancer drugs, reduce the risk of clinical trial failure, and save R&D time and costs (Fig. 3).

## Current status of PDTO biobanks

**Figure 4:**
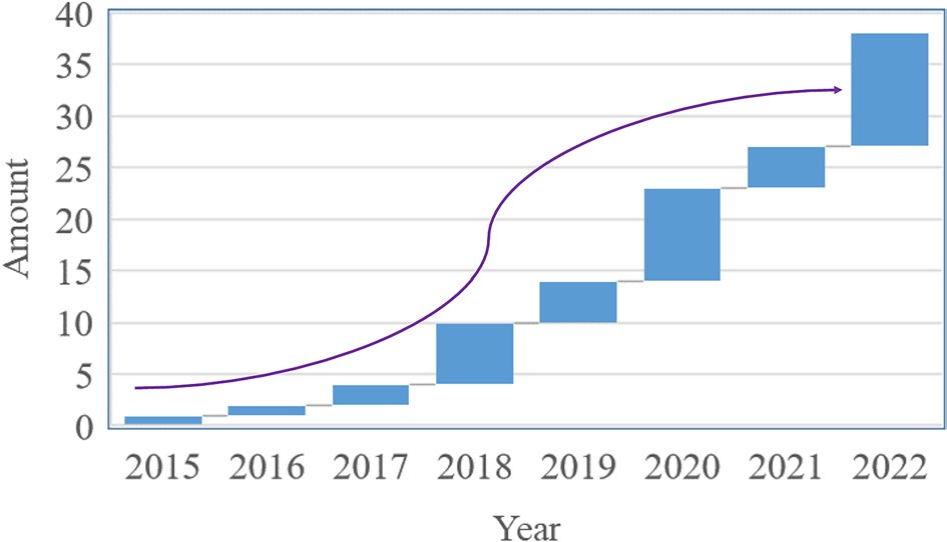
Temporal trends of biobanks establishment.

**Figure 5:**
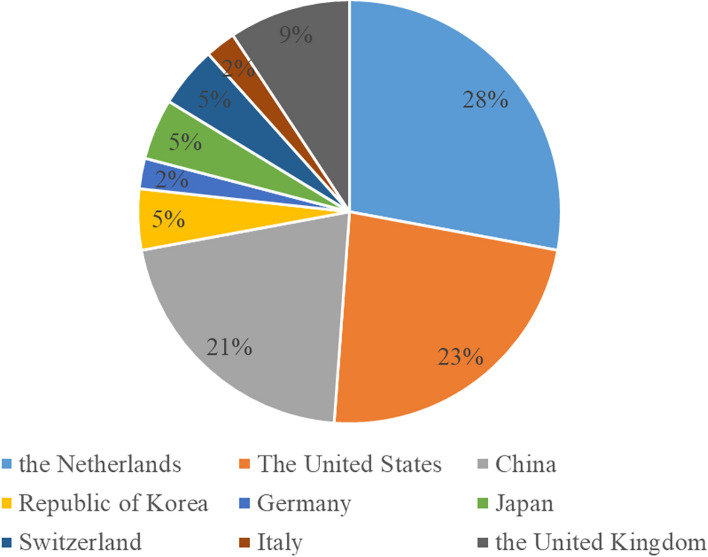
National distribution of PDTO biobanks.

**Figure 6:**
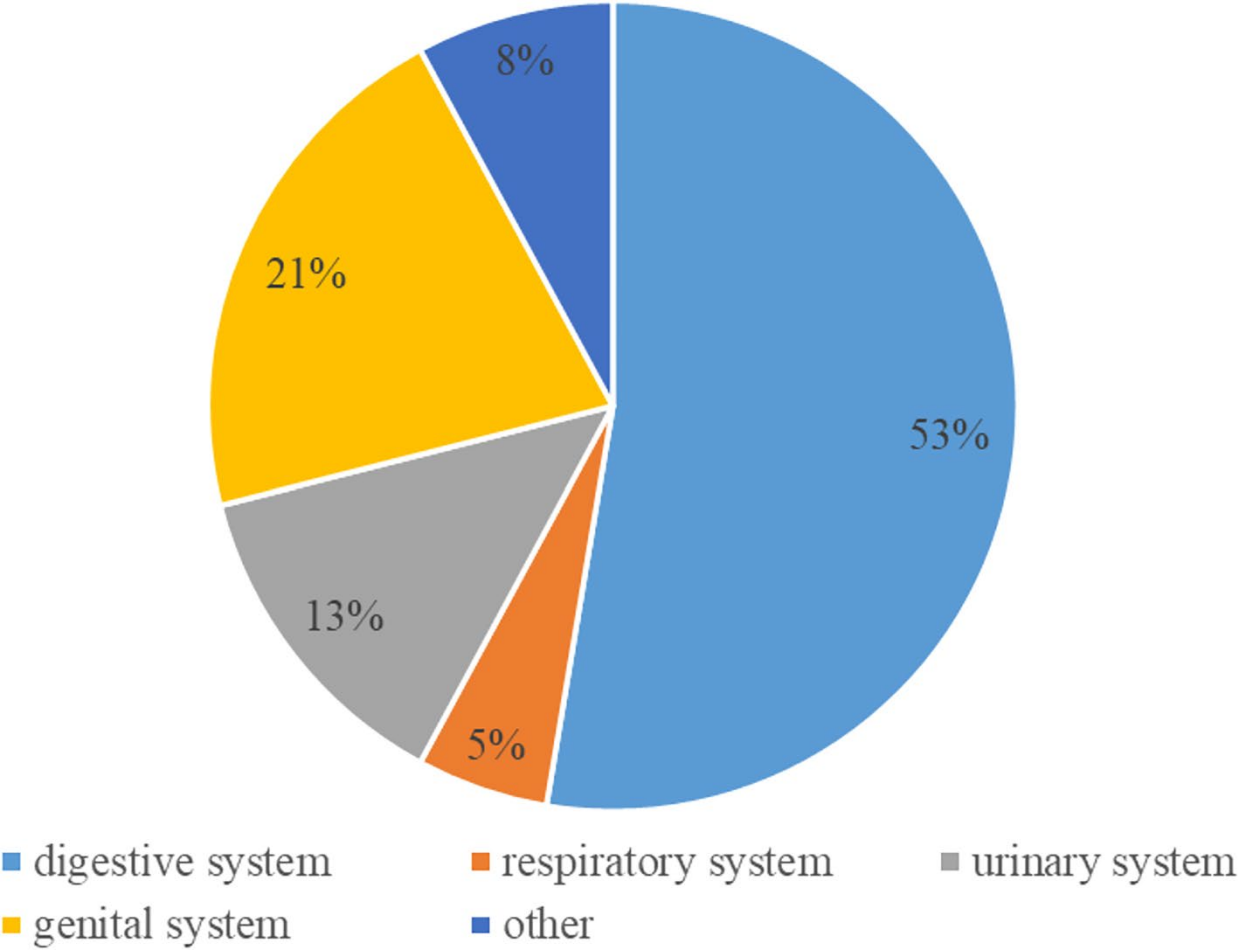
Distribution of organ systems in PDTO biobanks.

**Figure 7:**
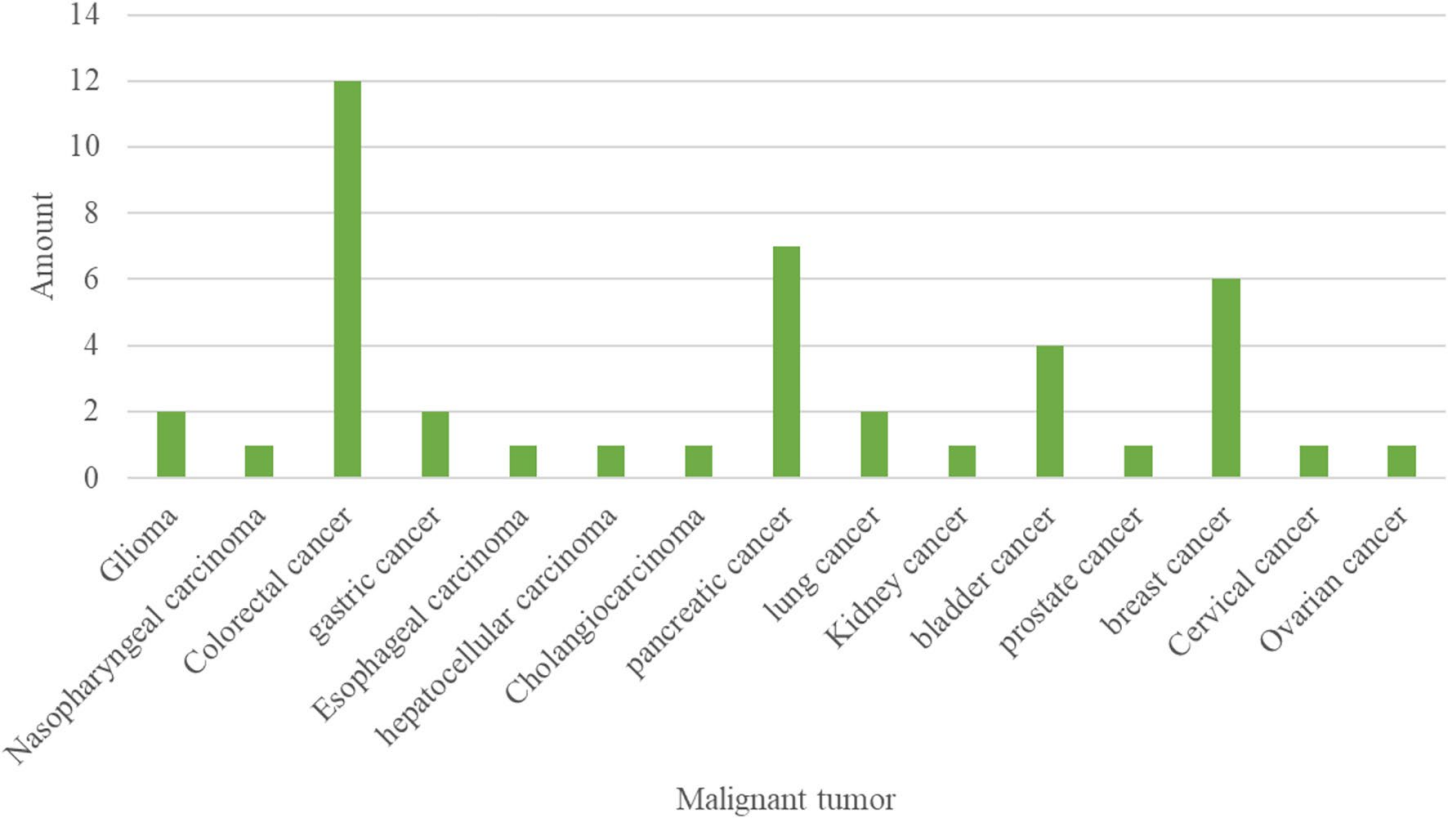
Status of organoid biobanks by tumor species.

**Figure 8:**
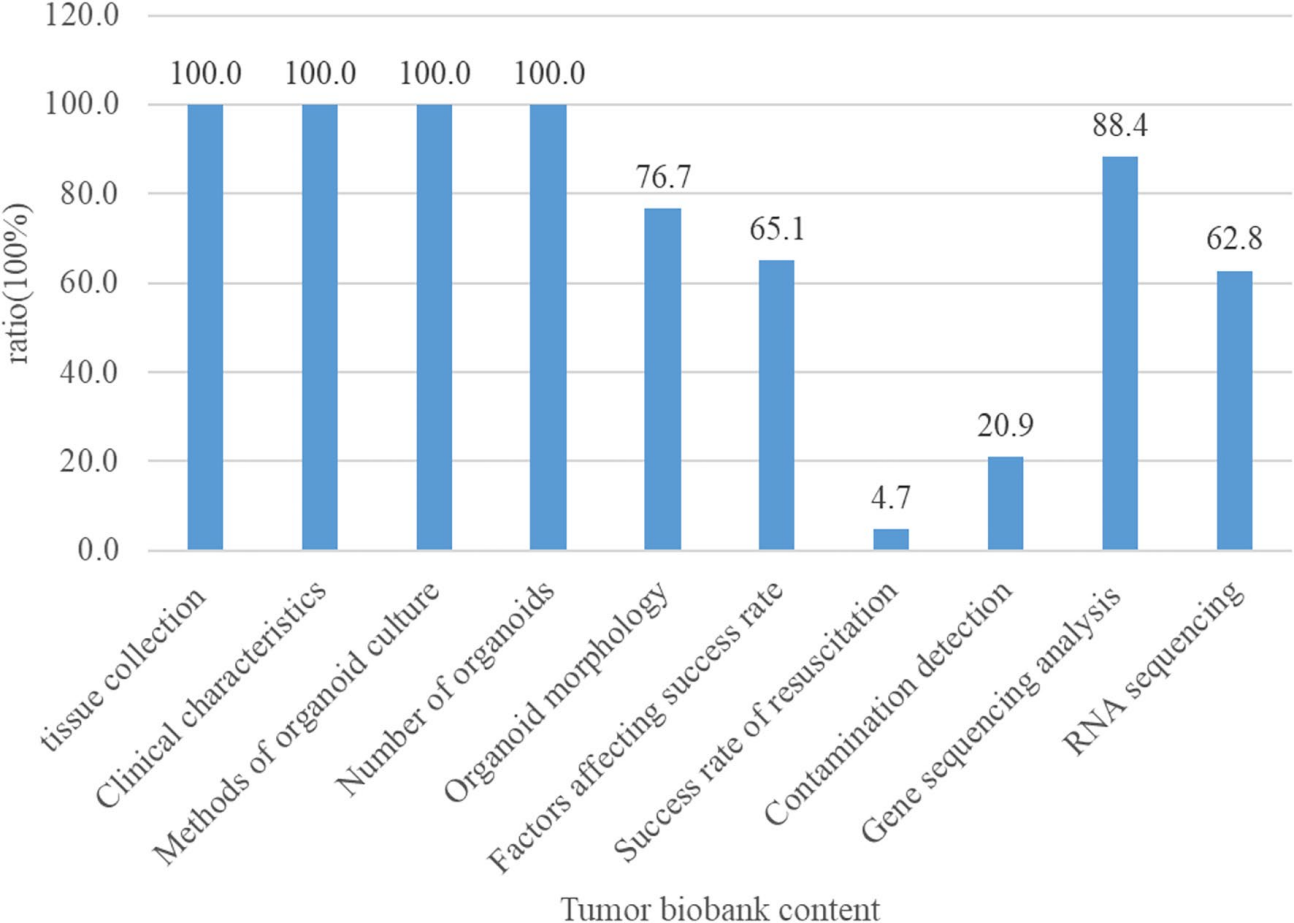
Common contents of PDTO biobanks.

At present, we have established 43 tumor organoid biobanks (Additional file 1), 20 of which are digestive malignant tumors, 2 of which are respiratory malignant tumors, 5 of which are urinary system tumors, and 8 of which are genital system tumors, 3 of which are head and neck malignant tumors, in addition to covering 5 comprehensive biobanks. An increasing number of PDTO biobanks were established (Fig. 4). This reflected the increasing maturity of organoid culture techniques. Among all the countries that have built PDTO biobanks (Fig. 5), the Netherlands, the United States and China had the top three culture number of PDTO biobanks. In our results, we found that digestive and genital system built the most biobanks (Fig. 6). Among them, colorectal cancer, pancreatic cancer, breast cancer, glioma, and bladder cancer were the most established tumors (Fig. 7), with the most mature culture technology. Common contents of established organoid biobanks were shown in Fig. 8. According to our statistical analysis, all PDO biobanks contained information on tissue sources and types, clinical characteristics and the number of organoid lines. Compared with organoid morphology, culture success rate, and verification experiments, organoid biobank resuscitation and contamination detection were poorly documented. Contamination detection was only recorded in 20.9% reports, and success rate for resuscitation was even lower, at only 4.7%. The necessary information should be reported for the PDTO biobanks.

## Opportunities and challenges in establishing PDTO biobanks

PDTOs can be used for cancer modeling, drug screening and development, biobanking, and more. So far, oncologists have made great efforts to establish organoid biobanks by developing various efficient organoid culture systems. Although organoids have developed by leaps and bounds, they still face lots of challenges. First, most PDTOs only contain the epithelial layer, lack the physiological microenvironment (such as muscle layer, stromal cells, immune cells, vascular endothelial cells, and nervous system)^119^. Therefore, there are limitations in the research of immunotherapy drugs and anti-vascular drugs. But this can be overcomed by technologies such as co-culture and organ-on-a-chip^120^. By integrating multiple micro-organs into various microchambers connected to each other^121^, scientists can study the interaction of cancer multi-organ metastasis and detect adverse drug events^122^. Co-culture systems can partially replicate tumor microenvironment^123–127^ by adding additional microorganisms and cells. In addition, 3D bioprinting technology^128,129^ can be used to develop complex organoid models. Skardal et al.^130^ combined 3D printed heart and liver organoids with lung organoids, and revealed the cardiotoxicity of bleomycin. Even if PDTOs can be transplanted into immunodeficient mice to create an internal microenvironment, there are certain limitations due to species differences. Despite the technical challenges exist, patient-derived immune cells and hematopoietic stem cells can be transplanted into immunodeficient mice to create humanized mice to compensate for this shortcoming^131,132^.

Second, organoids should be cultured within a specific matrix. However, Matrigel and BME are derived from mouse sarcoma, and there may be batch variations, which will interfere with the accuracy and repeatability of the experiment. Curvello et al. developed collagen-nanocellulose hydrogels to support organoid growth^133^. Extracellular matrices and supplemental factors are expensive, which may limit wide-scale application^134^. Hydrogels provide an economical and sustainable alternative matrix for PDTOs. Hirokawa et al.^135^ established intestinal PDOs and PDTOs through low-viscosity matrix suspension culture. The advent of hydrogels will help reduce the cost of organoid culture.

Fujii et al.^136^ found that different subtypes of colon cancer organoids require different media. However, when culturing PDTOs, the genetic background is often uncertain. Different combinations of growth factors are required to improve the success culture rate of different tumor types. In addition, organoid culture systems vary widely among research teams, which may raise questions about the consistency of results. Karen et al.^137^ cultured ovarian cancer organoids under low-Wnt conditions, while another research group reported another protocol involving the addition of neuregulin 1^33^. The uniformity and accuracy of organoid research results will depend on standard experimental procedures and analyses agreed by experts in the future^138^.

At present, the ethical issues involved in PDTOs are also an inevitable challenge in establishing PDTO biobanks. Lensink et al.^139^ suggested that researchers should maintain a viable and sustainable research environment. The popularity of organoids has helped increase patients' willingness, but there is also concern that patients expect too much. Patients were highly willing to participate in organoid research^140^, and they also attached great importance to their right to withdraw tissues and organoids. In addition, if drug screening results differ significantly from guidelines, how clinicians use these results to guide clinical practice is an issue that deserves a careful consideration. From this point, the establishment of PDTO biobanks not only faces the problems of organoid culture itself, but also needs to solve ethical issues.

## Conclusion

PDTOs can better summarize the key characteristics and functions of parental tumor, ushering in a new era of precision medicine. At present, organoid biobanks of various tumors have been established, which provide reliable models for basic and clinical researches. In future, solving the uniformity of organoid technical standards and a good quality supervision system is crucial to the establishment of a safe, accurate and convenient PDTO biobank, which should attract extensive attention from researchers.

## Supplementary Information

Supplementary Information.

## Data Availability

The datasets generated and/or analyzed during the current study are available from the corresponding author on reasonable request.
